# Effectiveness of a Web-Based Solution-Focused Brief Chat Treatment for Depressed Adolescents and Young Adults: Randomized Controlled Trial

**DOI:** 10.2196/jmir.3261

**Published:** 2014-05-29

**Authors:** Jeannet Kramer, Barbara Conijn, Pien Oijevaar, Heleen Riper

**Affiliations:** ^1^Trimbos Institute (Netherlands Institute of Mental Health and Addiction)UtrechtNetherlands; ^2^Developlay BV, Publishing media for childrenUtrechtNetherlands; ^3^Department of Clinical Psychology and EMGO InstituteVU UniversityAmsterdamNetherlands; ^4^Innovation IncubatorLeuphana UniversityLueneburgGermany

**Keywords:** depression, randomized controlled trial, Internet, Solution Focused Brief Therapy, young adults

## Abstract

**Background:**

Up to 9% of young people suffer from depression. Unfortunately, many in need of help remain untreated. The Internet offers anonymous ways to help depressed youth, especially those who are reluctant to search for help because of fear of stigma.

**Objective:**

Our goal was to evaluate the effectiveness of an individual chat treatment based on Solution-Focused Brief Therapy (SFBT) to young individuals aged 12-22 years with depressive symptoms by comparing it to a waiting list control group.

**Methods:**

For this study, 263 young people with depressive symptoms were randomized to the Web-based SFBT intervention, PratenOnline, or to a waiting list control condition. The chat treatment was delivered by trained professionals. Groups were compared on depressive complaints as measured by the Center for Epidemiologic Studies Depression Scale (CES-D) after 9 weeks and 4.5 months. For the chat group only, changes in depressive symptoms at 7.5 months after baseline were explored.

**Results:**

The experimental SFBT condition (n=131) showed significantly greater improvement than the waiting list condition (n=132) in depressive symptoms at 9 weeks and 4.5 months on the CES-D, with a small between group effect size at 9 weeks (*d=*0.18, 95% CI -0.10 to 0.47) and a large effect size at 4.5 months (*d=*0.79, 95% CI 0.45-1.08). The percentage of participants showing a reliable and clinically significant change in depression was significantly larger for the SFBT intervention at 4.5 months only (28.2% vs 11.4% for the waiting list, *P*<.001, number needed to treat*=*6). At 7.5 months, the SFBT group showed further improvements. However, results have to be considered carefully because of high attrition rates.

**Conclusions:**

The Web-based SFBT chat intervention of PratenOnline was more effective than a waiting list control group in reducing depressive symptoms, and effects were larger at follow-up then at post-treatment. More studies are needed to find out if outcomes will be replicated, especially for those younger than 18 year old.

**Trial Registration:**

Netherlands Trial Register: NTR 1696; http://www.trialregister.nl/trialreg/admin/rctview.asp?TC=1696 (Archived by WebCite at http://www.webcitation.org/6DspeYWrJ).

## Introduction

### Background

Depression is among the most common mental health problems in young people. About 5.6% of youth aged 13-18 [1], and 9% of those aged 18-24 years [2] suffer from depression. Depression early in life can have serious implications on social, educational, and family functioning and is an important predictor of suicidal behavior [3].

Despite the high prevalence of depression in youth and the possible serious implications on their lives, depression in young people is often unrecognized and undertreated [4]. Young people are not inclined to seek help for depression, and referral to treatment at mental health services is a bridge too far for most of them [5]. Perceived stigma and concern about family member responses are important barriers [6]. The reluctance of many depressed young people to engage with mental health services [7] highlights the importance of low threshold and easily accessible interventions. The Internet offers such an opportunity. The anonymity of the Internet reduces fear of stigma [8] and fits well into the “digital lifestyle” of young people.

An increasing number of Web-based services and interventions are available for children, adolescents, and young adults ranging from self-help materials to online treatments. Research on youth and young adults indicates that Web-based interventions can be effective in reducing depressive complaints [9,10]. However, outcomes of randomized controlled trials have had mixed results, with some showing better outcomes compared to a waiting list for males only [11], the whole sample [12], or compared to an active control condition [13]. Some did not show significant differences between group effects when compared to a waiting list [14] or to an active treatment control group [15]. Most studies focused on Cognitive Behavioral Therapy or Problem Solving Therapy, but none of the studies on Web-based treatments are based on Solution-Focused Brief Therapy (SFBT) [16]. SFBT shifts the focus away from problem formation and problem resolution, to participants’ future goals, strengths, and resiliencies. In SFBT, a professional collaborates with the client to look for solutions to obtain goals and strongly stresses the client’s autonomy and competencies to achieve them. SFBT is a widely used therapeutic approach in coaching, couples therapy, and psychotherapy. According to several meta-analyses and reviews, it has positive effects in a broad range of settings and problem areas [17-21]. In the most recent and comprehensive review, five studies focus on depression as an outcome [21]. One study focused on mildly depressed college students [22] and found that one session of SFBT was as effective as one session of interpersonal therapy with a significant decrease in depressive symptoms. Other studies on SFBT with adult populations showed that SFBT was related to a reduction of depressive symptoms over time, and comparable outcomes to short-term psychodynamic therapy [23], past-focused treatment [24], common factors therapy [25], and a treatment based on the Hazeldon model in a group of substance abusers [26]. None of these studies were about Web-based interventions.

### Current Study

In this paper, we present the results of a trial on a Web-based anonymous SFBT chat intervention for depressed adolescents and young adults aged 12-22 years. The trial was started after a pilot study showed promising results: a positive evaluation by participants and a decrease from pre- to post-intervention with a large effect size (*d*=1.32) [27]. The trial was conducted to find out if the SFBT chat intervention was effective in reducing depressive symptoms compared to a waiting list control group. To the best of our knowledge, no randomized controlled trial has been published on the effectiveness of Web-based treatments based on SFBT, in adolescents or adults.

## Methods

### Study Design

A randomized controlled trial with two parallel groups was conducted to evaluate the effectiveness of the PratenOnline chat intervention (Chat) by comparing it to a waiting list control group (WL). This study was registered with the Netherlands Trial Register (NTR 1696). Ethical approval was granted by an independent medical ethics committee (Centrale Commissie Mensgebonden Onderzoek, CCMO No. NL25219.097.08).

### Study Population

Participants were young people with depressive symptoms who fulfilled the following criteria: (1) 12-22 years of age, (2) had access to a computer and Internet, (3) had a CES-D score of 22 or higher (the cut-off to detect possible cases of depression among adolescents) [28], (4) gave informed consent, and (5) completed a baseline questionnaire. Applicants were excluded when there was an indication of suicidal ideation with intent and plan as measured with an item of the Quick Inventory of Depressive Symptomatology-Self rated (QIDS-SR) [29].

### Recruitment

Participants for the study were recruited through articles in newspapers, and banners and links placed on relevant websites for youth and on Facebook. Young people interested in participating were referred to the PratenOnline website for information about the study and to fill in a screening questionnaire to check the criteria for involvement. Those aged 12-17 years with a CES-D score of 22 or higher were invited to fill in a Web-based informed consent form and baseline questionnaire. Candidates younger than 18 years also needed written parental consent. After inclusion, participants were automatically randomized to one of two conditions: the PratenOnline chat intervention (Chat) or the waiting list control condition (WL). Random allocation was automated by a computer program without interference of the intervention supervisor or researcher. Participants were informed by email of their allocation, and the Chat participants were asked to schedule their first chat session via the intervention website. During the study, the PratenOnline chat intervention was exclusively accessible for applicants participating in the study. Blinding of participants, therapists, and researchers was not possible due to the design of this study. During the trial, participants in both conditions were allowed to seek additional help if they wished.

### The Intervention

The intervention is a brief Web-based Solution-Focused synchronous chat intervention for young people aged 12-22 with depressive symptoms called PratenOnline (Talking online) [30]. It is offered by a mental health care foundation for youth in the Netherlands (Stichting Jeugdriagg Noord Holland Zuid) and has been online since 2004. The chat consists of individual real-time chat sessions with a trained health care professional in a secured chat room. During the sessions, SFBT techniques [31] are used by the therapist, starting with asking the “miracle” question (ie, a question that asks the patient to envision and describe how the future will be different when the problem is gone), setting goals, looking for strengths or solutions, keeping the focus on what is going well or better, giving compliments, looking for exceptions to the problem, and asking the client to indicate on scales from 1-10 what progress is made in obtaining goals. At the end of each chat session, the participant decides if their intervention goal has been reached. If not, a new chat session is scheduled with the therapist. The intervention is accessible anonymously, without cost for participants and available during weekdays, (late) nights, and weekends. After registration, the participant can choose three possible dates for a chat with a therapist. The confirmation of the chat can be found after logging in to the personal mailbox on the intervention website. No reminders could be sent to an email address outside of this secured environment because of anonymity reasons. The chat intervention follows the principles of SFBT [16] and is performed according to the guidelines of the European Brief Therapy Association (EBTA; the EBTA Solution Focused Practice Definitions [32]). A chat session takes about one hour. The intention is to keep the number of chats limited to five, but more sessions are delivered when needed.

### Conditions: The Waiting List

The waiting list (WL) group did not receive access to the chat intervention. They could participate after the waiting period of 4.5 months.

### Assessments

Assessments took place before randomization (baseline, t0), 9 weeks (t1), and 4.5 months after baseline (t2). At 7.5 months after baseline (t3), a last follow-up measurement took place, exclusively for participants in the Chat condition, to measure effects at longer term. All assessments consisted of self-reported Web-based questionnaires and took about 15 minutes to complete. Email reminders were sent after 7 days if necessary. To stimulate response, participants received a voucher of €10 for each completed questionnaire (t1, t2, and t3).

### Primary Outcome Measure: Depressive Symptoms

Symptoms of depression in the past week were assessed with the 20-item CES-D [33,34]. The total score ranges from 0-60, with higher scores reflecting more depressive symptoms. Construct validity and reliability of the CES‐D are well established for the paper-and-pencil, computerized, and Internet versions [28,35]. In our study, Cronbach alpha ranged from .75-.81.

### Additional Measures

At baseline, demographic characteristics (ie, sex, age, educational level, daily activity, living situation, ethnic background), duration of the psychological complaints (ie, how long the current complaints had been present), and professional help received ever before and at present were assessed. At t1, professional help and use of medication were measured. Attendance of chats was automatically measured by client Web statistics.

### Power

Originally, the trial was powered to detect clinically significant health gains expressed as a standardized effect size of a medium size (difference between groups of at least *d*=0.40) in a one-sided test with an alpha of .05 and a power (1-beta) of .80. The results reported in this paper, however, are based on more conservative two-tailed tests.

### Analyses

All analyses were performed on the intention-to-treat sample with missing values imputed. The expectation-maximization (EM) method was used to impute missing data. It imputes values by maximum-likelihood estimation using the observed data in an iterative process [36]. *T* tests, chi-square tests, and non-parametric Mann-Whitney U tests (**P*<.*05) were used to assess whether the randomization had resulted in two comparable groups at baseline and whether any differential loss to follow-up had occurred. Logistic regression was used (backward method) to find predictors of completing questionnaires and attending chats in the Chat condition (0=no chats, 1=one or more chats).

Change scores based on EM imputation were used to analyze differences between groups at 9 weeks and 4.5 months (a positive score means improvement). Variables on which conditions differed significantly at baseline were regarded as relevant confounders when causing a change of 10% in the regression coefficient for condition when added to the regression model [37]. While no relevant confounders were found, results of independent samples *t* tests are shown.

As attrition was rather high, sensitivity analyses were run to study the robustness of the estimates of EM imputation, using the multiple imputation Predictive Mean Matching method (PMM) in Stata (creating 100 datasets). PMM combines the standard linear regression and the nearest-neighbor imputation approaches. Predictors of outcome and missingness were taken into account to impute missing CES-D outcomes. Analyses were performed in a multiple imputation framework. Also data of completers of questionnaires were analyzed.

Magnitudes of intervention effects were estimated using Cohen’s *d* [38]. Within group effect sizes were first calculated for each condition separately ((M_t0_–M_t1_) / SD_t0_) and subsequently the between group effect size delta *d* by subtracting the effect size of the WL group from that of the Chat group. For Cohen’s *d*, an effect size of 0.2 to 0.3 may be regarded as a small effect, around 0.5 as a medium effect, and 0.8 to infinity as a large effect.

The proportion of participants showing reliable and clinically significant improvement [39] was defined by an improvement of 5 points in combination with a score lower than 22 on the CES-D (cut-off based on Cuijpers et al, 2008) [28]. Differences between groups were tested with chi-square tests. The number needed to treat was calculated as 1/success rate difference [40].

The change from baseline to 7.5 months (t3) was explored in the Chat condition only by means of a one sample two-sided *t* test, comparing the change in CES-D from baseline to 7.5 months.

The analyses were performed using SPSS (version 19.0) and Stata (version 11.1).

## Results

### Participants

**Figure 1:**
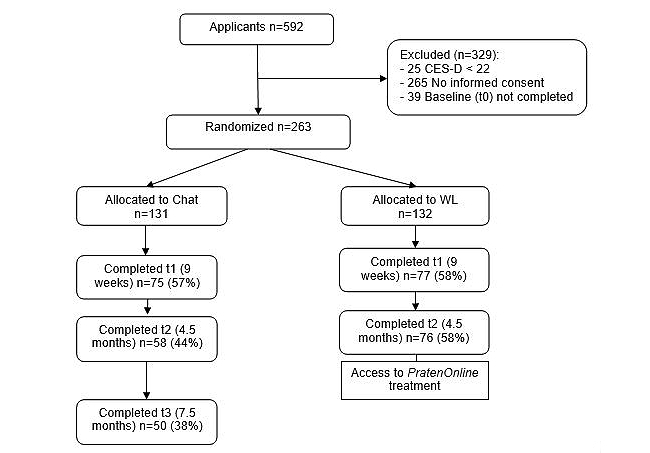
Flow of participants.

Participants were recruited from August 9, 2009, until January 24, 2010. Most participants were recruited via Internet (520/592, 87.8%). Others applied on advice of a person (61/592, 10.3%) or after reading about it in a magazine or newspaper (11/592, 1.9%). Figure 1 shows the flow of participants through the trial.

Of the 592 young people who applied, 263 (44.4%) were included in the study. Reasons for non-inclusion were lack of informed consent (265/329, 80.5%), not completing the t0 questionnaire (39/329, 11.9%), and a CES-D depression score lower than 22 (25/329, 7.6%). Only 10 (3.8%) participants included were between 12 and 17 years of age (five assigned to each arm). Of the 253 applicants between 12 and 17 years of age, 243 were excluded, either because they did not return their parents’ consent (227/243, 93.4%) or had a CES-D score lower than 22 (16/243, 6.6%).

### Demographic Characteristics Participants

Baseline demographic, psychosocial, and clinical characteristics are shown in Table 1. Most participants were female (207/263, 78.7%). Over two thirds was still in school or studying (187/263, 71.1%). There were no differences between groups at baseline. Also there were no differences between groups in professional help received at t1 (χ^2^_1_=0.30, *P*=.59) and t2 (χ^2^_1_=0.07, *P*=.79) or in the use of antidepressant medication at t1 (χ^2^_1_=0.15, *P=*.70) and t2 (χ^2^_1_=0.02, *P=*.89).

**Table 1:** Baseline characteristics (n=263).

Characteristics		Chat n=131	WL n=132	All N=263	Statistics
Female, n (%)		104 (79.4)	103 (78.0)	207 (78.7)	χ^2^_1_=0.07, *P*=*.*79
Age, mean (SD)		19.4 (1.6)	19.6 (1.8)	19.5 (1.7)	*t*_261_=0.48, *P*=.48
**Age groups, years, n (%)**					χ^2^_1_=0.00, *P*=*.*99
	12-17	5 (3.8)	5 (3.8)	10 (3.8)	
	18-22	126 (96.2)	127(96.2)	253 (96.2)	
**Education level** ^a^ **, n (%)**					χ^2^_2_=2.75, *P*=*.*25
	Low	75 (57.3)	78 (59.1)	153 (58.2)	
	Middle	50 (38.2)	42 (31.8)	92 (35.0)	
	High	6 (4.6)	12 (9.1)	18 (6.8)	
**Daily activity, n (%)**					χ^2^_2_=0.57, *P*=*.*75
	Student (high school)	91 (69.5)	96 (72.7)	187 (71.1)	
	Paid job	20 (15.3)	20 (15.2)	40 (15.2)	
	Other	20 (15.3)	16 (12.1)	36 (13.7)	
**Living situation, n (%)**					χ^2^_3_=0.66, *P*=*.*88
	With parents	82 (62.6)	78 (59.1)	160 (60.8)	
	With partner	18 (13.7)	17 (12.9)	35 (13.3)	
	Alone	15 (11.5)	18 (13.6)	33 (12.5)	
	With others	16 (12.2)	19 (14.4)	35 (13.3)	
Had professional help before, n (%)		68 (51.9)	67 (50.8)	135 (51.3)	χ^2^_1_=0.04, **P*=.*85
Had professional help at baseline, n (%)		26 (19.8)	18 (13.6)	44 (16.7)	χ^2^_1_=1.82. **P*=.*18
Ethnic background^b^, n (%)		19 (14.5)	12 (9.1)	31 (11.8)	χ^2^_1_=1.85, **P*=.*17
Duration psychological complaints in years, n (%)		0.70 (1.1)	0.52 (0.8)	0.61 (1.0)	*t*_261_=-1.57, **P*=.*12
CES-D depression score, mean (SD)		39.5 (8.6)	39.7 (7.1)	39.6 (7.9)	*t*_251.8_=0.26, **P*=.*79

^a^Education: lower=primary education or lower general secondary education, middle=intermediate vocational or high school, high=higher vocational education or university.

^b^Non-western immigrants when one or both parents is born in Africa, Latin America, or Asia (including Turkey and excluding Indonesia, Japan, and Dutch East Indies).

### Attrition

A total of 42.2% (111/263) of the participants did not complete t1, and 49.0% (129/263) did not complete t2. The groups did not differ at t1 in returning completed questionnaires (χ^2^_1_=0.03, **P*=.*86). Some statistically significant differences at baseline were detected between participants who completed measurements and those who did not. At t1, non-completers were more often males (χ^2^_1_=6.51, **P*=.*01), lived with their parents more often (χ^2^_1_=7.17, **P*=.*007), had a longer history of mood problems (χ^2^_1_=4.85, **P*=.*03). At t2, non-completers were more often in the Chat group (χ^2^_1_=4.65, **P*=.*03), were less often at school or studying (χ^2^_1_=4.42, **P*=.*04), and had at baseline more thoughts about suicide (χ^2^_1_=6.01, **P*=.*01). These results indicate that loss to follow-up was not completely at random. Assessment at 7.5 months (t3) was not completed by 61.8% (81/131) in the Chat group. At t3, non-completers differed from completers in depressive complaints at baseline: non-completers more often had a score of 40 or higher on the CES-D (χ^2^_1_=5.35, **P*=.*02).

### Effect of the Intervention: Primary Outcome, Depressive Symptoms

**Figure 2:**
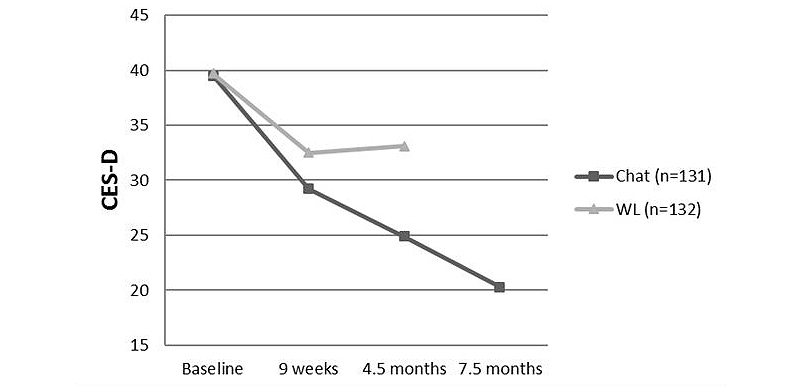
Means on CES-D per measurement for Chat (n=131) and WL (n=132) (EM-imputed data).

The results for the CES-D outcomes for the intention-to-treat sample are depicted in Table 2, and mean CES-D scores per measurement are shown in Figure 2. The results of *t* tests show that depressive symptoms decreased significantly more in the Chat condition from baseline to 9 weeks with a small between group effect size (*d=*0.18, 95% CI -0.10 to 0.47) and from baseline to 4.5 months with a large between group effect size of *d=*0.79 (95% CI 0.45-1.08). The sensitivity analyses with PMM imputed data showed significant differences between groups only at 4.5 months, with effect sizes a bit lower than the EM outcomes, but of the same magnitude being again small at 9 weeks and large at 4.5 months. Results of the analyses including only completers of questionnaires show significant differences between groups at 4.5 months, with again a large effect size in favor of the Chat condition. No significant differences were found at 9 weeks.

**Table 2:** Means and estimates for depression score (CES-D) at 9 weeks (t1) and 4.5 months (t2) follow-up: intention-to-treat (EM imputation) and completers only (CO) analysis.

	Chat			WL			Between group test of change scores			
	N	Mean (SD)	*d*	N	Mean (SD)	*d*	Δ*d* ^a^	*t*	df	*P* value, 2-sided
T0	131	39.49 (8.58)		132	39.74 (7.13)					
T1 EM	131	29.20 (10.66)	1.20	132	32.51 (9.68)	1.01	0.18	2.70	261	.007
T2 EM	131	24.86 (8.51)	1.72	132	33.09 (9.69)	0.93	0.79	6.39	261	<.001
T1 PMM	131	29.78 (14.41)	1.13	132	32.78 (12.95)	0.98	0.16	1.78	151.8	.08
T2 PMM	131	26.36 (14.81)	1.53	132	32.99 (13.26)	0.95	0.58	3.45	134.9	.001
T1 CO	74	29.49 (12.11)	1.07	77	33.00 (10.95)	0.94	0.14	1.53	149	.13
T2 CO	58	24.66 (10.87)	1.57	76	33.37 (11.32)	0.82	0.75	3.66	132	<.001

^a^ *Δd* between group effect size.

### Depressive Symptoms at 7.5 Months in the Chat Group

At 7.5 months (t3), the mean CES-D score of the Chat group was 20.31 (SD 10.06) showing a mean change of 19.18 points since baseline (*t*_130_=17.40, *P*<.001) and a large within group effect size of *d=*1.60 from baseline to 7.5 months. Figure 2 shows a graphical representation of CES-D outcomes.

### Reliable and Clinical Change

At 9 weeks, 22.1% (29/131) participants in the Chat condition and 13.6% (18/132) in the WL condition showed reliable and clinically significant change. This difference between conditions was not significant (χ^2^_1_=3.24, *P*<.07). The number needed to treat was 11.7. At 4.5 months, 28.2% (37/131) in the Chat group and 11.4% (15/132) in the WL group showed a reliable and clinically significant change. This between-group difference was significant (χ^2^_1_=11.81, *P*<.001) and yielded a number needed to treat of 6.0. At 4.5 months, still 92 (70.2%) participants in the Chat group and 116 (87.9%) in the WL group scored 22 or higher on the CES-D, indicating they might still have clinical depression.

### Sessions Attended and Outcome

**Figure 3:**
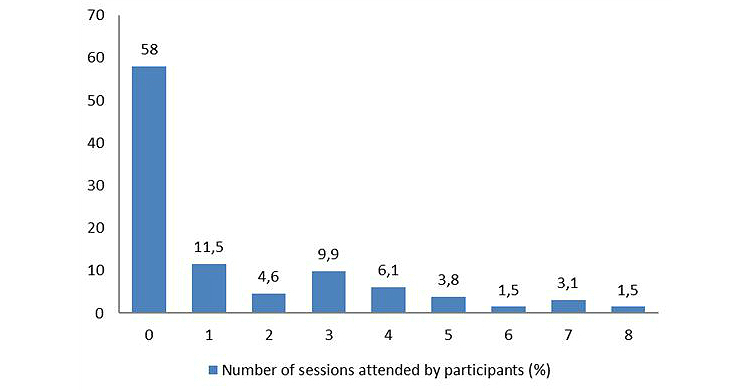
Number of chat sessions attended by percentages of participants (n=131).

The number of sessions attended by the subjects in the Chat condition is shown in Figure 3. The mean number of chats was 1.36 (SD 2.08), with on average 4.27 weeks (SD 6.27) between the first and last chat session (range 0-27 weeks).

According to client Web statistics, 55.7% (73/131) logged into the appointment system and 42.0% (55/131) actually had one or more chats, and 58.0% (76/131) did not have any chats. At t1 and t2, not all participants in the Chat condition had completed their therapy (n=14 had chat sessions after t1 and n=6 after t2). There were no significant differences in changes in depressive symptoms between those who attended at least 1 chat session and those who had none (at 9 weeks: *d=*1.22 vs *d=*1.19, *t*_129_=-0.16, **P*=.*88; at 4.5 months: *d=*1.59 vs *d=*1.79, *t*_129_=0.87, **P*=.*39). When those who did not chat in the Chat condition were compared to the WL group, the non-chatters (Chat) did not differ significantly from the WL group at 9 weeks (*d*=1.19 vs *d=*1.01, *t*_187.8_=-1.07, *P*=.29), but they did show better outcomes at 4.5 months (*d=*1.79 vs *d=*0.93, *t*_206_=-4.50, **P*<.*001).

## Discussion

### Principal Results

The present study shows considerable improvements in depressive symptoms in both the Chat group and the waiting list group over time, but more so for the SFBT chat group, indicating it was more effective than the waiting list control condition. Between group effect sizes were small at 9 weeks (*d=*0.18) but increased after 4.5 months (*d=*0.79). At 7.5 months, the Chat condition showed further improvements. The more favorable outcomes for the Chat condition were also reflected in the significantly larger proportion of participants showing a reliable and clinically significant improvement for the Chat condition at 4.5 months, but not yet at 9 weeks. Despite the improvements, a large group had not fully recovered at 4.5 months and more than 70% of the chat intervention group still experienced depressive complaints above the cut-off (CES-D≥22) indicating they might still be struggling with depression.

### Comparison With Other Work

There are no Web-based studies on SFBT interventions to compare our results with, but effect sizes reported for offline SFBT in the meta-analysis of Kim (2008) [19] are in the range of *d*=0.13 to *d*=0.26. These studies were based on only a few studies with limited numbers of participants. The study by Van der Zanden et al (2012) [12] is the most similar in target group and delivery of intervention: a chat intervention. Van der Zanden et al studied the effectiveness of a Web-based structured 6-session group chat intervention, guided by professionals, for young people aged 16-25 years, with mild to moderate depressive complaints. The intervention consisted of 6 structured sessions of CBT. As in our study, the group chat intervention proved more effective than the waiting list condition, with a large between group effect size at 3 months (*d*=0.94). The proportion of participants with a reliable and clinically significant change (based on the same criteria to define CES-D changes) was 56%, which is twice as high as in our study (28%). This difference in proportion of “recovered” participants might relate to the lower baseline level of depressive complaints in Van der Zanden’s study (mean 32.5 vs mean 39.6 in our study). If there are fewer depressive complaints to start with, less improvement is needed over time to reach the same threshold of 22 on the CES-D. In both studies, it was found that participants who did not chat displayed equal improvements to those who did and that non-chatters improved more at follow-up than the waiting list group. An explanation for this effect might be that not starting or discontinuing treatment could mean that participants experienced improvement and thought treatment was no longer necessary, while participants with more persistent depressive complaints started or continued treatment with hope of obtaining relief. This might especially be the case in treatments where treatment sessions are not fixed but determined by the needs of patients [41].

When compared to outcomes of face-to-face treatments for adolescents found in a meta-analytic review [42], the pre-post intervention effect size for face-to-face treatments was nearly the same (*d*=1.23) as the effect size found in our study at 9 weeks (*d*=1.20), and the between group follow-up effect size of *d*=0.64 of face-to-face treatments was even a bit smaller than the effect size found in our study (*d*=0.79) at 4.5 months. This shows that the PratenOnline chat intervention has effects that match and even exceed those found in a meta-analysis on face-to-face treatments for adolescents.

### Limitations

In our study, attrition (ie, dropping out of the study) was high. This is a phenomenon often observed in studies of Web-based interventions both among adults and youngsters [43,44]. This may have to do with the low threshold that makes it easy to start, but also easy to stop. A consequence of the high attrition rates is that a substantial number of missing observations had to be imputed, and this may have influenced outcomes. Since we do not know why attrition took place, it is hard to say if we have overestimated or underestimated the effect of the chat intervention. Results of our study, therefore, need to be considered with caution. However, the sensitivity analyses and completers only analyses show similar results to the EM-imputed data analyses, providing some confidence in the validity of the conclusions.

In our study, only 42% of those who had access to the Chat intervention of PratenOnline made use of it, although 56% had made an appointment for a chat. Limited adherence is not uncommon in Web-based interventions [45,46], and different factors can be of influence. These can be personal factors like a lack of motivation or time, an improvement or deterioration in mood, a need for face-to-face contact, or technological factors like computer problems or a lack of Internet skills [47].

In the daily chat practice of PratenOnline, the age group of 12-17 years old is highly represented. In the trial, however, only 10 (3.8%) participants in that age group were included. The major bottleneck to participate for this age group was the parental consent that had to be provided by both parents in a written consent form. Other studies also had problems with recruitment because of parental consent [12,48], making it difficult to get a handle on the effectiveness of Web-based interventions for those younger than 18 years. This also means we have to be careful in generalizing the results to young people aged 12-17 years.

In our study, a waiting list control condition was used. This might have effected the magnitude of the between group effect sizes. As Clarke et al pointed out, “the between group effect size is not just a function of the potency of the experimental intervention but is also a function of the magnitude of change observed in the control condition” (2009, page 231) [13]. Effect sizes tend to be lower when a comparison with a “strong active” intervention control condition is used [49]. But for progression of research, both studies with no-active control conditions and studies with active control conditions are necessary [13]. And although the between group effect sizes might be affected by the control group being a waiting list, the within group effects of the Chat intervention are not expected to do so that much, and these underpin the potential effects of the intervention.

### Future Research Directions

As far as we know, this is the first study on a brief Web-based Solution-Focused Intervention for young people with depressive complaints. Despite the limitations of the present study, our findings indicate that adolescents and young adults with depressive symptoms can profit from access to the Web-based SFBT chat treatment. Studies in this field are few, but this one contributes to the evidence that Web-based interventions can be effective. However, because of the limitations of the study, more research is needed to find out if outcomes will be replicated. Especially for young people under the age of 18, more evidence is needed for the effectiveness of Web-based SFBT. To make such studies successful, the major impediment to include this age group needs to be tackled: the parental consent.

## Multimedia Appendix 1

CONSORT-EHEALTH checklist V1.6.2 [50].

## Abbreviations

CES-D: Center for Epidemiologic Studies Depression Scale
CO: completers only
EM: expectation-maximization
PMM: predictive mean matching
QIDS-SR: Quick Inventory of Depressive Symptomatology—Self rated
SFBT: Solution-Focused Brief Therapy
WL: waiting list
