# Supplementary material for: Effectiveness of a Web-Based Solution-Focused Brief Chat Treatment for Depressed Adolescents and Young Adults: Randomized Controlled Trial
Source: J Med Internet Res. 2014 May 29;16(5):e141. doi: 10.2196/jmir.3261 (PMC4062279; doi:10.2196/jmir.3261)
Supplement: Supplementary file 1 [file jmir_v16i5e141_app1.pdf]

CONSORT-EHEALTH Checklist V1.6.2 Report

(based on CONSORT-EHEALTH V1.6), available at [http://tinyurl.com/consort-ehealth-v1-6].

3261

Date completed

1/19/2014 4:56:58

by

Jeannet Kramer

The effectiveness of a web-based Solution Focused Brief Chat Treatment for depressed adolescents and young adults: a randomized controlled trial.

TITLE

1a-i) Identify the mode of delivery in the title

"The effectiveness of a web-based Solution Focused Brief Chat Treatment for depressed adolescents and young adults: a randomized controlled trial."

1a-ii) Non-web-based components or important co-interventions in title

"web-based"

1a-iii) Primary condition or target group in the title

The effectiveness of a web-based Solution Focused Brief Chat Treatment for depressed adolescents and young adults: a randomized controlled trial.

ABSTRACT

1b-i) Key features/functionalities/components of the intervention and comparator in the METHODS section of the ABSTRACT

"To evaluate the effectiveness of an individual chat treatment based on Solution-Focussed Brief Therapy (SFBT) to young individuals aged 12 – 22 years with depressive symptoms by comparing it to a waiting list control group."

1b-ii) Level of human involvement in the METHODS section of the ABSTRACT

"The chat treatment was delivered by trained professionals"

1b-iii) Open vs. closed, web-based (self-assessment) vs. face-to-face assessments in the METHODS section of the ABSTRACT

No.

1b-iv) RESULTS section in abstract must contain use data

"The experimental SFBT condition (n=131) showed significantly greater improvement than the Waiting list condition (n = 132) in depressive symptoms at 9 weeks and 4.5 months on the CES-D with a small between group effect size at 9 weeks (d=0.18, 95% CI -0.10 - 0.47) and a large effect size at 4.5 months (d=0.79, 95% CI 0.45 - 1.08). The percentage of participants showing a reliable and clinically significant change in depression was significantly larger for the SFBT intervention at 4.5 months only (28.2% vs 11.4% for the waiting list, P<.001, NNT=6). At 7.5 months the SFBT group showed further improvements. Results have to be considered carefully, though, because of high attrition rates."

1b-v) CONCLUSIONS/DISCUSSION in abstract for negative trials

No negative results, so no need to discuss this.

INTRODUCTION

2a-i) Problem and the type of system/solution

"Most studies focused on Cognitive Behavioral Therapy or Problem Solving Therapy, but none of the studies on web-based treatments are based on Solution Focused Brief Therapy (SFBT) [15]. SFBT shifts the focus away from problem formation and problem resolution, to participant's future goals, strengths and resiliencies. In SFBT a professional collaborates with the client to look for solutions to obtain goals and strongly stresses the client's autonomy and competences to achieve them. SFBT is a widely used therapeutic approach in coaching, couples therapy and psychotherapy"

2a-ii) Scientific background, rationale: What is known about the (type of) system

"and according to several meta-analyses and reviews it has positive effects in a broad range of settings and problem areas [16,17,18,19,20]. In the most recent and comprehensive review five studies focus on depression as an outcome [20]. One study focused on mildly depressed college students [21] and found that one session of SFBT was as effective as one session of Interpersonal therapy with a significant decrease in depressive symptoms. Other studies on SFBT with adult populations showed that SFBT was related to a reduction of depressive symptoms over time, and comparable outcomes to short-term Psychodynamic therapy [22], Past-focused treatment [23], Common factors therapy [24] and a treatment based on the Hazeldon model in a group of substance abusers [25]. None of these studies were about web-based interventions."

METHODS

3a) CONSORT: Description of trial design (such as parallel, factorial) including allocation ratio

"In the current study we present the results of a trial on a web-based anonymous SFBT chat intervention for depressed adolescents and young adults aged 12 to 22 years. The trial was started after a pilot study showed promising results: a positive evaluation by participants and a decrease from pre- to post-intervention with a large effect size (d = 1.32) [26]. The trial was conducted to find out if the SFBT chat intervention was effective in reducing depressive symptoms compared to a waiting list control group. "

3b) CONSORT: Important changes to methods after trial commencement (such as eligibility criteria), with reasons

No changes.

3b-i) Bug fixes, Downtimes, Content Changes

No, we do not have information about this. The chat was delivered by professionals in a chatroom and we are not aware of any downtime.

4a) CONSORT: Eligibility criteria for participants

"Participants were young people with depressive symptoms who fulfilled the following criteria: 1) 12 to 22 years of age, 2) having access to a computer and Internet, 3) having a CES-D score of 22 or higher, the cut-off to detect possible cases of depression among adolescents [27]; 4) informed consent and 5) a completed baseline questionnaire. Applicants were excluded when there was an indication of suicidal ideation with intent and plan as measured with an item of the QIDS-SR [28]. "

4a-i) Computer / Internet literacy

We did not ask for this, as young people are very much digital in their lifestyle.

4a-ii) Open vs. closed, web-based vs. face-to-face assessments:

"Participants for the study were recruited through articles in newspapers, and banners and links placed on relevant websites for youth and on Facebook. "

4a-iii) Information giving during recruitment

"Young people interested in participating were referred to 'www.pratenonline.nl' for information about the study and to fill in a screening questionnaire. Those who met the study inclusion criteria were invited to fill in an informed consent form and baseline questionnaire".

4b) CONSORT: Settings and locations where the data were collected

"All assessments were self-reported web-based questionnaires"

4b-i) Report if outcomes were (self-)assessed through online questionnaires

"All assessments were self-reported web-based questionnaires"

4b-ii) Report how institutional affiliations are displayed

Only the name 'www.PratenOnline.nl' was mentioned in advertisements. No other institutions.
5) CONSORT: Describe the interventions for each group with sufficient details to allow replication, including how and when they were actually administered
5-i) Mention names, credential, affiliations of the developers, sponsors, and owners
"... called 'PratenOnline' (Talking online). It is offered by a mental health care foundation for youth in the Netherlands (Stichting Jeugdriagg Noord Holland Zuid) and is online since 2004. "
5-ii) Describe the history/development process
No, because if is a chat intervention with a professional, this is not relevant.
5-iii) Revisions and updating
No, because if is a chat intervention with a professional, this is not relevant.
5-iv) Quality assurance methods
Not relevant.
5-v) Ensure replicability by publishing the source code, and/or providing screenshots/screen-capture video, and/or providing flowcharts of the algorithms used
No, because if is a chat intervention with a professional, this is not relevant.
5-vi) Digital preservation
Because if is a chat intervention with a professional, it is not relevant to provide screenshots.

The URL is mentioned: "Talking online, www.pratenonline.nl"
5-vii) Access
"The intervention is accessible anonymously, without cost for participants and available during weekdays, (late) nights and weekends. After registration, the participant can choose three possible dates for a chat with a therapist. "
5-viii) Mode of delivery, features/functionalities/components of the intervention and comparator, and the theoretical framework
This was not an automated intervention, but a chat. So not so relevant for this study.
Synchronous is mentioned: "The intervention is a brief web-based Solution-Focused synchronous Chat intervention"
5-ix) Describe use parameters
"The intention is to keep the number of chats limited to five, but more sessions are delivered when needed"
5-x) Clarify the level of human involvement
"The chat consists of individual real-time chat sessions with a trained healthcare professional in a secured chat room."
5-xi) Report any prompts/reminders used
For the intervention: "No reminders could be send to an e-mail address outside of this secured environment, because of anonymity reasons."
For assesssments: 'E-mail reminders were sent after 7 days if necessary. "
5-xii) Describe any co-interventions (incl. training/support)
No co-intervention involved.
6a) CONSORT: Completely defined pre-specified primary and secondary outcome measures, including how and when they were assessed
"Primary outcome measure: Depressive Symptoms
Symptoms of depression in the past week were assessed with the 20-item Center for Epidemiological Studies Depression Scale (CES-D) [30,31]. The total score ranges from 0 to 60, with higher score reflecting more depressive symptoms. Construct validity and reliability of the CES-D are well established for the paper-and-pencil, computerized and Internet versions [32, 27]. In our study, Cronbach's alpha ranged from .75 to .81.

Additional measures
At baseline, demographic characteristics (sex, age, educational level, daily activity, living situation, ethnic background), duration of the psychological complaints and lifetime and current professional help for psychological problems were assessed. Professional help and use of medication was also measured at t1 and t2. Attendance of chats was automatically measured by client web statistics."
6a-i) Online questionnaires: describe if they were validated for online use and apply CHERRIES items to describe how the questionnaires were designed/deployed
"Construct validity and reliability of the CES-D are well established for the paper-and-pencil, computerized and Internet versions [32, 27]"
6a-ii) Describe whether and how “use” (including intensity of use/dosage) was defined/measured/monitored
"Attendance of chats was automatically measured by client web statistics. "
6a-iii) Describe whether, how, and when qualitative feedback from participants was obtained
Not included in this study.
6b) CONSORT: Any changes to trial outcomes after the trial commenced, with reasons
Not relevant.
7a) CONSORT: How sample size was determined
7a-i) Describe whether and how expected attrition was taken into account when calculating the sample size
Originally, the trial was powered to detect clinically significant health gains expressed as a standardized effect size of a medium size (difference between groups of at least d =.0.40) in a one-sided test with an alpha of 0.05 and a power (1-beta) of 0.80.
7b) CONSORT: When applicable, explanation of any interim analyses and stopping guidelines
Not applicable.
8a) CONSORT: Method used to generate the random allocation sequence
"Random allocation was automated by a computer program without interference of the intervention supervisor or researcher. "
8b) CONSORT: Type of randomisation; details of any restriction (such as blocking and block size)
None mentioned.
9) CONSORT: Mechanism used to implement the random allocation sequence (such as sequentially numbered containers), describing any steps taken to conceal the sequence until interventions were assigned
Random allocation was automated by a computer program without interference of the intervention supervisor or researcher. "
10) CONSORT: Who generated the random allocation sequence, who enrolled participants, and who assigned participants to interventions
"Random allocation was automated by a computer program without interference of the intervention supervisor or researcher. "
11a) CONSORT: Blinding - If done, who was blinded after assignment to interventions (for example, participants, care providers, those assessing outcomes) and how
11a-i) Specify who was blinded, and who wasn’t
No blinding.

11a-ii) Discuss e.g., whether participants knew which intervention was the “intervention of interest” and which one was the “comparator”
The participants knew because of the waiting list what the intervention and the comparator condition was.
11b) CONSORT: If relevant, description of the similarity of interventions
The interventions were not similar (waiting list control).
12a) CONSORT: Statistical methods used to compare groups for primary and secondary outcomes
"Change scores based on EM imputation were used to analyze differences between groups at 9 weeks and 4.5 months (a positive score means improvement). Variables on which conditions differed significantly at baseline were regarded as relevant confounders when causing a change of 10% in the regression coefficient for condition when added to the regression model [34]. While no relevant confounders were found, results of independent samples t-tests are shown. "
12a-i) Imputation techniques to deal with attrition / missing values
"The expectation-maximization (EM) method was used to impute missing data. It imputes values by maximum-likelihood estimation using the observed data in an iterative proces"
12b) CONSORT: Methods for additional analyses, such as subgroup analyses and adjusted analyses
"As attrition was rather high, sensitivity analyses were run to study the robustness of the estimates of EM imputation, using the multiple imputation Predictive Mean Matching method (PMM) in Stata (creating 100 data sets). PMM combines the standard linear regression and the nearest-neighbor imputation approaches. Predictors of outcome and missingness were taken into account to impute missing CES-D outcomes. Analyses were performed in a multiple imputation framework. Also data of completers of questionnaires were analyzed."

RESULTS

13a) CONSORT: For each group, the numbers of participants who were randomly assigned, received intended treatment, and were analysed for the primary outcome
These are shown in the flow chart.
13b) CONSORT: For each group, losses and exclusions after randomisation, together with reasons
The flow charts shows the losses after randomisation. Reasons are not availble, while they were not provided by the participants.
13b-i) Attrition diagram
"The number of sessions attended by the subjects in the chat condition is shown in figure 3"
14a) CONSORT: Dates defining the periods of recruitment and follow-up
No exact dates, only the description of this is delivered in the methods section.
"Assessments took place before randomization (baseline, t0), 9 weeks (t1) and 4.5 months after baseline (t2). At 7.5 months after baseline (t3) a last follow-up measurement took place, exclusively for participants in the Chat condition, to measure effects at longer term. "
14a-i) Indicate if critical “secular events” fell into the study period
None.
14b) CONSORT: Why the trial ended or was stopped (early)
Not applicable.
15) CONSORT: A table showing baseline demographic and clinical characteristics for each group
Available in table 1. "Table 1. Baseline characteristics (n = 263). "
15-i) Report demographics associated with digital divide issues
Demographics are mentioned, but nog associated with digital divide issues.
16a) CONSORT: For each group, number of participants (denominator) included in each analysis and whether the analysis was by original assigned groups
16-i) Report multiple “denominators” and provide definitions
"The number of sessions attended by the subjects in the chat condition is shown in figure 3.'
16-ii) Primary analysis should be intent-to-treat
"The results for the CES-D outcomes for the intention-to-treat sample are depicted in table 2 and mean CES-D scores per measurement are shown in figure 2. "
17a) CONSORT: For each primary and secondary outcome, results for each group, and the estimated effect size and its precision (such as 95% confidence interval)
"The results of t-tests show that depressive symptoms decreased significantly more in the Chat condition from baseline to 9 weeks with a small between group effect size (d=0.18, 95% CI -0.10 - 0.47) and from baseline to 4.5 months with a large between group effect size of d=0.79 (95% CI 0.45 - 1.08)."
17a-i) Presentation of process outcomes such as metrics of use and intensity of use
See also earlier: "The number of sessions attended by the subjects in the chat condition is shown in figure 3"
17b) CONSORT: For binary outcomes, presentation of both absolute and relative effect sizes is recommended
For recovered participants:
"At 9 weeks, 22.1% (29/131) participants in the Chat condition and 13.6% (18/132) in the WL condition showed reliable and clinically significant change. This difference between conditions was not significant ($\chi^2(1)= 3.24$, P<.07). The number needed to treat was 11.7. At 4.5 months 28.2% (37/131) in the Chat group and 11.4% (15/132) in the WL group showed a reliable and clinically significant change. This between-group difference was significant ($\chi^2(1)= 11.81$, P<.001) and yielded a number needed to treat of 6.0. At 4.5 months still 92 (70.2%) participants in the Chat group and 116 (87.9%) in the WL group scored 22 or higher on the CES-D, indicating they might still be having a clinical depression. "
18) CONSORT: Results of any other analyses performed, including subgroup analyses and adjusted analyses, distinguishing pre-specified from exploratory
Sensitivity analyses: "The sensitivity analyses with PMM imputed data showed significant differences between groups only at 4.5 months, with effect sizes a bit lower than the EM outcomes, but of the same magnitude being again small at 9 weeks and large at 4.5 months. Results of the analyses including only completers of questionnaires show significant differences between group at 4.5 months, with again a large effect size in favor of the Chat condition. No significant differences were found at 9 weeks."
18-i) Subgroup analysis of comparing only users
Completers only:
"Results of the analyses including only completers of questionnaires show significant differences between group at 4.5 months, with again a large effect size in favor of the Chat condition. No significant differences were found at 9 weeks."
19) CONSORT: All important harms or unintended effects in each group
None
19-i) Include privacy breaches, technical problems
Was not relevant for this study.
19-ii) Include qualitative feedback from participants or observations from staff/researchers
Not mentioned in this article: lack of space in the article and also only limited information available.

DISCUSSION

20) CONSORT: Trial limitations, addressing sources of potential bias, imprecision, multiplicity of analyses

20-i) Typical limitations in ehealth trials

"Limitations
In our study attrition – drop out of the study - was high. This is a phenomenon often observed in studies about web-based interventions both among adults and youngsters [39,40]. This may have to do with the low threshold that makes it easy to start, but also easy to stop. A consequence of the high attrition rates is that a substantial number of missing observations had to be imputed and this may have influenced outcomes. As we do not know why attrition took place, it is hard to say if we have over- or underestimated the effect of the chat intervention. Results of our study, therefore, need to be considered with caution. However, the sensitivity analyses and completers only analyses show similar results as the EM-imputed data analyses, providing some confidence in the validity of the conclusions.
In our study only 42% of those who had access to the Chat intervention of PratenOnline, made use of it, although 56% had tried to make an appointment for a chat. Limited adherence is not uncommon in web-based interventions [41,42] but it is unclear what the impact of non-attendance in treatments like the Chat treatment of PratenOnline means. In our study we found that participants who did not chat, displayed equal improvements as those who did, and non-chatters improved more at follow-up than the waiting list group. The same effect was found in the study of Van der Zanden et al. (2012) [11]. An explanation for this effect might be that discontinuation of treatment could mean that participants experienced improvement and thought treatment was no longer necessary, while participants with more persistent depressive complaints continued treatment with hope on obtaining relief. This might especially be the case in treatments where treatment sessions are not fixed but determined by the needs of patients [43].
In the daily chat practice of PratenOnline, the age group of 12-17 years old is highly represented. In the trial, however, only 10 (3.8%) participants in the age group of 12 to 17 years old were included. For this age group, the major bottleneck to participate was the parental consent that had to be provided by both parents in a written consent form. Also other studies had problems with recruitment because of the parental consent [44,11], making it difficult to get a good grip on the effectiveness of web-based interventions for those younger than 18 years. This also means we have to be careful in generalizing the results to young people aged 18 to 22 years.
In our study a waiting list control condition was used. This might have effected the magnitude of the between group effect sizes. As Clarke et al. (2009, page 231) [12] pointed out: 'the between group effect size is not just a function of the potency of the experimental intervention but is also a function of the magnitude of change observed in the control condition'. Effect sizes tend to be lower when a comparison with a 'strong active' intervention control condition is used [45]. But for progression of research both studies with no-active control conditions and studies with active control condition are necessary [12]. And although the between group effect sizes might be effected by the control group being a waiting list, the within group effects of the Chat intervention are expected much less to do so, and these underpin the potential effects of the intervention."

21) CONSORT: Generalisability (external validity, applicability) of the trial findings

21-i) Generalizability to other populations

"This also means we have to be careful in generalizing the results to young people aged 18 to 22 years."

21-ii) Discuss if there were elements in the RCT that would be different in a routine application setting

Non mentioned.

22) CONSORT: Interpretation consistent with results, balancing benefits and harms, and considering other relevant evidence

22-i) Restate study questions and summarize the answers suggested by the data, starting with primary outcomes and process outcomes (use)

"The present study shows large improvements in depressive symptoms in both the Chat group and the waiting list group over time, but more so for the SFBT chat group, indicating it was more effective than the waiting list control condition. Between group effect sizes were small at 9 weeks (d=0.18) but became large after 4.5 months (d=0.79). At 7.5 months the Chat condition showed further improvements. The more favorable outcomes for the Chat condition were also reflected in the significantly larger proportion of participants showing a reliable and clinically significant improvement for the Chat condition at 4.5 months, but not yet at 9 weeks. Despite the improvements, at 4.5 months still a large group had not fully recovered and more than 70% of the chat intervention group still experienced depressive complaints above the cut-off (CES-D >= 22) indicating they might still be struggling with a depression. "

22-ii) Highlight unanswered new questions, suggest future research

"... more studies are needed to find out if outcomes will be replicated. Especially for young people below the age of 18 years old, more evidence is needed for the effectiveness of web-based SFBT. "

Other information

23) CONSORT: Registration number and name of trial registry

"Trial registration: Netherlands Trial Register: NTR 1696; "

24) CONSORT: Where the full trial protocol can be accessed, if available

Not available.

25) CONSORT: Sources of funding and other support (such as supply of drugs), role of funders

"The study was funded by a research grant from ZonMw (Netherlands Organization for Health Research and Development), grant no. 15700.2003. No funding bodies had any role in study design, data collection and analysis, decision to publish, or preparation of the manuscript."

X26-i) Comment on ethics committee approval

"Ethical approval was granted by an independent medical ethics committee (CCMO no. NL25219.097.08). "

x26-ii) Outline informed consent procedures

It was obtained online and offline. "Those who met the study inclusion criteria were invited to fill in an informed consent form and baseline questionnaire. Candidates younger than 18 years also needed written parental consent. "

X26-iii) Safety and security procedures

None mentioned.

X27-i) State the relation of the study team towards the system being evaluated

"Conflicts of Interest
Pien Oijevaar was one of the founders of the chat therapy 'Praten Online' in the Netherlands, but she did not derive financial income from the PratenOnline intervention. There are no competing interests."
